# Barriers to Bystander Intervention in Sexual Harassment: The Dark Triad and Rape Myth acceptance in Indonesia, Singapore, and United Kingdom

**DOI:** 10.1177/08862605211072150

**Published:** 2022-02-15

**Authors:** Minna Lyons, Gayle Brewer, Iona Bogle, Jorge Castro Caicedo, Monic Gaspar, Carissa Ghayda, Maya Huelin, Tan Wei Liang, Luna Centifanti

**Affiliations:** 1 4589 School of Psychology, Liverpool John Moores University, Liverpool, UK; 2 4591 School of Psychology, University of Liverpool, Liverpool, UK; 3 37580 Department of Psychology, National University of Singapore, Singapore

**Keywords:** cross-country, dark triad, bystander intervention, rape myths, sexual violence

## Abstract

Bystanders have an important role in preventing sexual violence, but they are often reluctant to intervene due to a range of barriers. In this study, we investigated relationships between the Dark Triad of personality (i.e. psychopathy, Machiavellianism and narcissism), rape myth acceptance and five bystander barriers. We addressed the paucity of research by collecting data from three countries (Indonesia, Singapore, and United Kingdom). In total, 716 University staff and students participated in an online survey. We found very few country-level differences in the correlations between the variables. In regression analyses, Machiavellianism and rape myth acceptance both had significant, positive relationships with failure to identify risk, failure to take responsibility, skills deficits and audience inhibition. Narcissism and psychopathy were significantly, negatively associated with audience inhibition and skills deficits. Findings indicate similarity in predictors of perceived barriers to bystander intervention across the three countries.

Bystanders have an important role in the prevention of sexual violence, with opportunities to intervene before, during, or after the event takes place (McMahon & Banyard, 2012). Nevertheless, victims report that in most cases prior to sexual assault, bystanders were present, but failed to take preventative action (Haikalis et al., 2018). Bystander inaction relates to multiple dispositional factors, including inaccurate myths around rape (e.g., Jozkowski et al., 2021; Lyons, Brewer, et al., 2021). There is a lack of knowledge of personality as a dispositional barrier, as well as a dearth of studies outside the United States (Labhardt et al., 2017). The present study addressed this gap by investigating rape myth acceptance, socially aversive personality traits (i.e., the Dark Triad) and bystander barriers in three countries (Indonesia, Singapore, and United Kingdom). Bystander intervention programmes have been effective in preventing sexual violence in American samples (Mujal et al., 2021), and our study has the potential to increase understanding of some of the barriers in countries outside the United States. In order to develop bystander workshops that encourage intervention prior to assault, it is important to investigate dispositional factors that form a barrier to intervention, as well as consider whether these vary across different countries.

Based on a situational model, Burn (2009) suggested five barriers to bystander inaction. Burn identified several intra and inter-personal factors that could hinder interventions at each of the five (i.e., failure to notice, failure to identify risk, failure to take responsibility, skills deficits and audience inhibition) steps. These steps are heavily influenced by the situation, characteristics of the bystander, their perceptions and their relationship to the victim and the perpetrator (e.g. Bennett et al., 2014; Burn, 2009; Pugh et al., 2016; Robinson et al., 2020).

First, bystanders may fail to notice the event because they are distracted (e.g., loud music), or their focus is elsewhere (e.g., on themselves or on other social interactions). Failure to notice has been identified as a large barrier in student populations where alcohol is often involved (Kania & Cale, 2021), and especially among individuals who have a personal history of sexual victimisation (Kistler et al., 2021). Other intrapersonal factors are lack of pro-social attitudes and low perceived control of situations (Bennett et al., 2014).

Second, even if bystanders do notice the event, they may not perceive it as high risk that is worthy of intervention. People often help if they are certain that a situation involves abuse (Bennett et al., 2014; Pugh et al., 2016), and may fail to look for more subtle cues indicating that a person is at risk. Individuals with lower levels of self-efficacy (Yule & Grych, 2020), and higher levels of benevolent sexism and rape myth acceptance (Yule et al., 2020) are more likely to perceive situations as low risk.

Third, bystanders may lack personal responsibility. Indeed, in a large sample of US university students, a large proportion of those who did not intervene reported the reason as the incident being “none of their business” (Hoxmeier et al., 2020). The lack of personal responsibility could be influenced by a number of factors, such as moral perceptions of the victim (e.g., promiscuity and intoxication), diffusion of responsibility, or individual characteristics of the bystander (Bennett et al., 2014; Pugh et al., 2016; Robinson et al., 2020; Yule et al., 2020). For instance, lack of pro-social attitudes, low perceived control (Bennett et al., 2014), benevolent sexism, and rape myth acceptance (Yule et al., 2020) have all been related to absence of personal responsibility.

Fourth, bystanders may feel that they lack skills and knowledge on how to act. This step is an important one, as intervention may happen only if the bystander feels safe, and confident about what to do (Bennett et al., 2014). Skills deficits seem to be a larger perceived barrier for women than for men (Yule & Grych, 2020; Yule et al., 2020). It has also been related to benevolent sexism in women (Yule et al., 2020).

Fifth, bystanders may fear negative consequences such as social evaluation or aggression from others. This indicates that individual characteristics (e.g., shyness or social anxiety) may stop some individuals from helping in sexual assault situations (Uhrig, 2018). Research has found that individuals who have poorer emotion regulation (Yule & Grych, 2020), and perceptions of low control in situations (Bennett et al., 2014) have more inhibitions in the presence of an audience.

One individual difference that has been consistently associated with many of these intervention barriers is rape myth acceptance (Kania & Cale, 2018; Labhardt et al., 2017; Lyons, Brewer, et al., 2021; Martini & De Piccoli, 2020). Rape myth acceptance is a widely studied idea in psychology, conceptualised as stereotypical attitudes around rape, including blaming the victim and excusing the perpetrator (Payne et al., 1999). Studies have related rape myths to reluctance to help the victims of harassment, potentially because of lower levels of empathy (Leone, Schipani-McLaughlin, et al., 2020), and higher proclivity to blame the victim (Martini & De Piccoli, 2020). More specifically, people higher in rape myth acceptance may fail to identify situations as high risk, and/or fail to take responsibility for intervening (Yule & Grych, 2020; Yule et al., 2020). Personality traits that relate to rape myth acceptance may be of particular importance when trying to understand barriers to bystander intervention.

Our focus here is on a socially malevolent personality constellation, the Dark Triad (i.e., narcissism, Machiavellianism, and psychopathy; Lyons, 2019). The three traits share a common feature of antagonism (Vize et al., 2020), but each trait also has unique aspects, for example: grandiosity and entitlement (narcissism); cynicism, tactical manipulation (Machiavellianism); and impulsivity and callousness (psychopathy; Lyons, 2019). These traits correlate positively with higher acceptance of rape myths (Jonason et al., 2017b; Lyons, Brewer, et al., 2021; Sanchez-Ruiz et al., 2021). To our knowledge, no research to date has investigated the Dark Triad with regards to the five bystander intervention barriers.

There are several reasons to expect an association between Dark Triad traits and perceived barriers to bystander intervention. First, bystander behaviour is facilitated by empathy (e.g., Yule et al., 2020), which is typically lower in those high in Dark Triad traits (e.g., Jonason et al., 2013; Pajevic et al., 2018). Second, the Dark Triad, especially psychopathy, is characterised by sexist and rape-enabling attitudes (Brewer et al., 2019; Gluck et al., 2020; Jonason et al., 2017b; Lyons, Brewer, et al., 2021; Navas, Maneiro, Cutrín, Gómez-Fraguela, & Sobral, 2020), which also are amongst the most important barriers to bystander action (Hook, Worthington, & Utsey, 2009; Yule & Grych, 2020; Yule et al., 2020). Third, a large body of research has demonstrated that the Dark Triad traits relate to higher incidences of perpetration of sexual coercion (Blinkhorn et al., 2015; Figueredo et al., 2015; Lyons et al., 2020; Muñoz et al., 2011), which also constitutes a barrier to bystander intervention (Lyons, Brewer, et al., 2021; Yapp & Quayle, 2018). In the present study, we investigated how the Dark Triad and rape myth acceptance related to the five barriers to intervention identified by Burn (2009).

As well as adding to the Dark Triad literature, we expanded bystander barrier research by considering a number of countries. To date, almost all studies on barriers to bystander intervention have been conducted in universities in the United States (Labhardt et al., 2017), with very few examples from other parts of the world (although see Kania & Cale, 2018 for a study in Australia; Hennelly et al., 2019 for a study in the United Kingdom; Lyons, Brewer, et al., 2021 for a study in Ecuador; and Kamimura et al., 2016 for a comparison between United States and Asian countries). There are likely to be country-related differences in bystander behaviour, potentially linked to factors such as gender equality and social norms around gender (e.g., Glick et al., 2000). Here, we added to the sparse cross-national literature on bystander behaviour by investigating bystander barriers in Indonesia, Singapore and United Kingdom.

The countries in our study differ in several dimensions, which makes it difficult to predict how bystander barriers may vary. For example, rape myth acceptance is more prevalent in world regions with higher gender inequality (e.g. Fakunmoju et al., 2021). Based on the gender equality index (Indonesia 85th, Singapore 54th, and United Kingdom 21st place globally; World Economic Forum, 2020), it would be expected that the UK participants identify fewer barriers to bystander intervention. However, the focus of the present study was not to compare countries on bystander barriers per se, but to investigate whether the correlations between personality, rape myth acceptance, and bystander barriers vary from one country to another. For instance, previous research has indicated that correlations between the Dark Triad and other variables (e.g., thinking styles; factors associated with risk-taking; and concerns for future) are similar across different countries and world regions (Jonason et al., 2020; Jonason et al., 2018). Thus, irrespective of country-level differences, we expected individual-level correlations between personality and bystander behaviours to be similar across the countries

In summary, we aimed to add to the sparse literature on barriers to bystander intervention in University staff and students in three countries (i.e., Indonesia, Singapore and the United Kingdom), taking into consideration aversive personality traits (i.e., the Dark Triad) and rape myth acceptance. The paucity of previous literature did not allow us to make specific predictions on how the predictor variables relate to the five different bystander barriers in our countries of interest. However, we made broad predictions that rape myth acceptance and the Dark Triad (especially psychopathy) should correlate positively with increased perceived barriers to act as a bystander, irrespective of the participants’ country.

## Method

### Participants

The survey, titled ‘Unwanted sexual attention in the cross-cultural context’, was advertised via snowball sampling on social media (i.e., Instagram, Twitter and Facebook). The team posted adverts in their social media pages, and asked their followers to complete and/or share the survey. The only inclusion criterion was that the participants had to be currently working or studying at a University. Because of the low number of postgraduates and staff members, this variable was not included in the analyses. Details for the sample are in Table 1.

**Table 1.** Demographic details of the sample.

Sample Characteristics	Indonesia *n* = 221	United Kingdom *n* = 343	Singapore *n* = 152
Mean age (*SD*)	20.05 (1.67)	20.97 (5.18)	21.97 (2.31)
Gender			
Male	49	37	53
Female	163	306	96
Other	3	0	1
Prefer not to say	6	0	2
University affiliation			
Undergraduate	194	302	143
Postgraduate	7	23	4
Staff	5	6	3
Prefer not to say	15	12	3

### Materials

The materials were in English, as University staff and students are expected to be proficient in this language in the countries in this study. In addition, the language of instruction in Universities in Singapore is English. For investigating bystander barriers, we utilised the Burn (2009) Bystander Barrier Scale. The questionnaire has five subscales calculated by summing and averaging the items on each subscale. Participants respond to all items on a 7-point Likert scale (1 = Strongly Disagree, 7 = Strongly Agree). The subscales are (i) Failure to Notice (one item; ‘*At a party or bar, I am probably too busy to be aware of whether someone is at risk for sexual assault*’), (ii) Failure to Identify Situation as High Risk (three items; e.g., ‘*In a party or bar situation, I think I might be uncertain as to whether someone is at risk for being sexually assaulted’,* α = .68), (iii) Failure to Take Intervention Responsibility (eight items; e.g., ‘*If I saw someone I did not know was at risk for being sexually assaulted, I would leave it up to his/her friends to intervene*’, α = .83), (iv) Failure to Intervene Due to a Skills Deficit (two items; e.g., ‘*Although I would like to intervene when a guy’s sexual conduct is questionable, I am not sure I would know what to say or do*’, α = .88) and (v) Failure to Intervene Due to Audience Inhibition (two items; e.g., ‘*I am hesitant to intervene when a man’s sexual conduct is questionable because I am not sure other people would support me*’, α = .78).

The Dark Triad was measured with the 27-item, 5-point (1 = Strongly Disagree, 5 = Strongly Agree) Short Dark Triad −3 scale (SD-3; Jones & Paulhus, 2014). The scale includes nine items per trait, with example items including ‘*People see me as a natural leader*’ (Narcissism, α = .70), ‘*It is not wise to tell your secrets*’ (Machiavellianism, α = .73) and ‘*People often say I am out of control*’ (Psychopathy, α = .71). The items were averaged to form an index of each Dark Triad trait.

For investigating rape myth acceptance, we used the 20-item, 7-point (1 = Strongly Disagree, 7 = Strongly Agree) version of the Illinois Rape Myth Acceptance Scale (IRMA; Payne et al., 1999). Example items include ‘*A woman who dresses in skimpy clothes should not be surprised if a man tries to force her to have sex*’, α = .93). The items were summed together to form an index of rape myth acceptance.

#### Procedure

On entering the online survey, participants read the participant information sheet, and provided consent. They were directed to a page asking demographic details, such as age, gender (male, female, other and prefer not to say) and country, and status in University (i.e. undergraduate, postgraduate, staff member and prefer not to say). The questionnaires were presented in this order: Short Dark Triad (SD-3), Bystander Barriers, Illinois Rape Myth Acceptance Scale (IRMA). Following completion, participants were directed to a debriefing page. The study received ethical approval by the Institutional Review Board.

## Results

The results for country and gender differences in the Short Dark Triad (SD-3), Bystander Barriers, Illinois Rape Myth Acceptance Scale (IRMA) are in Appendix 1. In Tables 2–4, we present the descriptive statistics and cross-correlations between all the variables for Indonesia, Singapore and the United Kingdom, respectively. Machiavellianism was significantly positively correlated with failure to take responsibility, regardless of country. Narcissism and psychopathy were negatively related to a skills deficit as a barrier, and these associations were significant in Indonesia and the United Kingdom. In Indonesia, narcissism correlated negatively with audience inhibition. Psychopathy had a weak, significant positive correlation with failure to take responsibility and failure to notice in Singapore. Rape myth acceptance was correlated positively in all countries with most of the barriers, with the exception of skills deficits. Age was related to few of the measures and was not included in subsequent analyses.

**Table 2.** Zero-Order Correlations and Descriptive Statistics for Age, Dark Triad Traits, Barriers to Bystander Intervention and Rape Myths in Indonesia.

	1	2	3	4	5	6	7	8	9	10
1. Age										
2.Machiavellianism	−.03									
3. Narcissism	.01	.23***								
4. Psychopathy	.12	.39***	.26***							
5. Failure to identify risk	.03	.06	−.23***	-.05						
6. Failure to take responsibility	−.01	.25***	.02	.01	.44***					
7. Skills deficits	−.16*	−.11	−.38***	−.28***	.44***	.26***				
8. Audience inhibition	−.07	.05	−.19**	−.13	.39***	.46***	.59***			
9. Failure to notice	.03	.07	−.04	.08	.42***	.15*	.23***	.22***		
10. Rape myth (log transformed)	−.07	.26***	.18**	.02	.13	.52***	.00	.23***	.14*	
*n*	221	221	221	221	221	221	220	221	221	216
*M*	20.10	3.40	2.92	2.53	3.75	3.47	4.58	3.90	3.66	2.29
*SD*	1.70	.49	.56	.54	1.10	.96	1.46	1.47	1.50	.70
Min	17	1.89	1.44	1.22	1.00	1.13	1.00	1.00	1.00	1.00
Max	30	4.89	4.78	4.00	6.00	6.13	7.00	7.00	7.00	4.46

*Note.* * *p* < .05, ** *p* < .01, *** *p* < .001*; descriptive statistics are based on non-transformed data*.

**Table 3.** Zero-Order Correlations and Descriptive Statistics for Age, Dark Triad Traits, Barriers to Bystander Intervention and Rape Myths in Singapore.

	1	2	3	4	5	6	7	8	9	10
1. Age										
2.Machiavellianism	.01									
3. Narcissism	.00	.30***								
4. Psychopathy	.23**	.39***	.40***							
5. Failure to identify risk	−.16	.07	−.10	.08						
6. Failure to take responsibility	.05	.32***	.06	.18*	.40***					
7. Skills deficits	−.28***	−.01	−.27***	−.16	.53***	.32***				
8. Audience inhibition	−.27***	.13	−.11	.05	.40***	.50***	.56***			
9. Failure to notice	.03	.15	.03	.18*	.53***	.30***	.31***	.26**		
10. Rape myth (log transformed)	.04	.25**	.00	.31***	.22**	.48***	.08	.19*	.22**	
*N*	152	152	152	152	152	152	152	152	152	147
*M*	22.00	3.19	2.48	2.17	4.09	3.58	5.13	3.96	3.40	2.07
*SD*	2.30	.55	.51	.52	1.18	1.01	1.53	1.73	1.54	.69
Min	18	1.67	1.00	1.00	1.00	1.00	1.00	1.00	1.00	1.00
Max	37	5.00	3.78	3.56	6.67	6.00	7.00	7.00	7.00	4.00

*Note.* * *p* < .05, ** *p* < .01, *** *p* < .001*; descriptive statistics are based on non-transformed data*.

**Table 4.** Zero-Order Correlations and Descriptive Statistics for Age, Dark Triad Traits, Barriers to Bystander Intervention and Rape Myths in the U.K.

	1	2	3	4	5	6	7	8	9	10
1. Age										
2.Machiavellianism	-−23***									
3. Narcissism	−.09	.32***								
4. Psychopathy	−.14**	.44***	.34***							
5. Failure to identify risk	.09	.11*	-.04	−.07						
6. Failure to take responsibility	.14**	.17**	.01	−.01	.44***					
7. Skills deficits	−.08	.04	−.15**	−.16**	.41***	.39***				
8. Audience inhibition	.2	.16**	-.02	-.04	.40***	.52***	.58***			
9. Failure to notice	.08	.07	.02	.03	.57***	.30***	.30***	.25***		
10. Rape myth (log transformed)	.03	.27***	.17**	.16**	.11*	.34***	.07	.17**	.15**	
*n*	343	343	343	343	343	343	343	343	343	335
*M*	21.00	2.81	2.57	2.13	3.45	2.64	4.31	3.13	3.12	1.46
*SD*	5.20	.55	.49	.53	1.19	.86	1.58	1.47	1.50	.41
Min	17	1.33	1.11	1.00	1.00	.88	1.00	1.00	1.00	1.00
Max	64	4.56	3.89	3.89	6.67	5.88	7.00	7.00	7.00	3.23

*Note.* * *p* < .05, ** *p* < .01, *** *p* < .001*; descriptive statistics are based on non-transformed data*.

We conducted five hierarchical regressions to test if Dark Triad traits statistically explained particular barriers to being an active bystander. Gender (0 = male, 1 = female, ‘other’ and ‘prefer not to say’ were left out in listwise deletions), country, and rape myths were entered in Step 1. Dark Triad traits were entered in Step 2. Finally, to test if the associations between Dark Triad traits and barriers would differ by country, Step 3 included three interaction terms (country*Machiavellianism, country*narcissism, country*psychopathy). None of these interactions were significant so we will not present these analyses further. All VIFs were less than 2.0, which suggested no problems with multicollinearity. We also tested assumptions of multivariate normality with Q-Q plots of residuals which showed samples close to the line except slight variations at the end points.

For failure to identify risks, Step 1 was significant, *F* (4, 681) = 13.62, *p* < .001. Higher levels of rape myths, and country were significant predictors (with more barriers in Singapore as opposed to the United Kingdom). At Step 2, Machiavellianism and narcissism were significant positive and negative predictors, respectively, explaining 2% of the variance, Δ*F* (3, 678) = 5.20, *p* < .001. The effects, however, were small; given the 95% confidence intervals were close to zero, especially for Machiavellianism (See Table 5).

**Table 5.** Hierarchical Regression Predicting Failure to Identify Risks by Country, Gender, Rape Myths and Dark Triad.

	Beta (SE)	β	95% CI		*R*^2^
Step 1			Lower	Upper	
Intercept	3.03 (.15)				.07***
Gender (1 = female)	.21 (.12)	.07	−.01	.15	
Country					
Indonesia – U.K.	.08 (.12)	.07	−.13	.27	
Singapore – U.K.	.52 (.13)	.44***	.23	.65	
Rape myth total - log	1.49 (.36)	.19***	.10	.29	
Step 2					Δ*R*^*2*^
Intercept	3.41 (.32)				.02***
Gender (1 = female)	.21 (.12)	.07	−.01	.15	
Country					
Indonesia – U.K.	.10 (.13)	.08	-.13	.29	
Singapore – U.K.	.42 (.13)	.35**	.14	.56	
Rape myth total - log	1.48 (.37)	.09***	.10	.29	
Machiavellianism	.20 (.09)	.10*	.01	.19	
Narcissism	−31 (.09)	−.14***	−.23	−.6	
Psychopathy	−.07 (.09)	−.03	.12	.05	

*Note.* * *p* < .05, ** *p* < .01, *** *p* < .001*; CI= confidence interval*.

For failure to take responsibility, Step 1 was significant, *R*^2^ = .35, *F* (4, 681) = 91.40, *p* < .001. Higher rape myth acceptance correlated with lower responsibility. In addition, Singaporean and Indonesian participants were more likely to fail to take responsibility than British participants were. Step 2 explained only 1% of the variance but this was significant, Δ*F* (3, 678) = 5.03, *p* = .002. Machiavellianism was uniquely associated with failure to take responsibility, but this effect was relatively small (see Table 6).

**Table 6.** Hierarchical Regression Predicting Failure to Take Responsibility by Country, Gender, Rape Myths and Dark Triad.

	Beta (SE)	β	95% CI		*R*^2^
Step 1			Lower	Upper	
Intercept	2.12 (.10)				.35***
Gender (1 = female)	.04 (.08)	.01	−.05	.08	
Country					
Indonesia – U.K.	.23 (.09)	.22*	.05	.39	
Singapore – U.K.	.51 (.09)	.51***	.33	.68	
Rape myth total - log	3.23 (.26)	.49***	.41	.56	
Step 2					Δ*R*^*2*^
Intercept	1.84 (.23)				.01**
Gender (1 = female)	.04 (.08)	.02	−.05	.08	
Country					
Indonesia – U.K.	.17 (.09)	.17	−.01	.34	
Singapore – U.K.	.44 (.09)	.43***	.25	.60	
Rape myth total - log	3.06 (.27)	.46***	.38	.54	
Machiavellianism	.26 (.07)	.15***	.07	.23	
Narcissism	−.07 (.06)	−.04	−.11	.03	
Psychopathy	−.11 (.07)	−.06	−.13	.01	

*Note.* * *p* < .05, ** *p* < .01, *** *p* < .001*; CI= confidence interval*.

For failure to intervene due to skills deficit, Step 1 was significant, *F* (4, 680) = 12.80, *p* < .001. Women were more likely to report skills deficits than men were, Singaporean participants reported this barrier to be greater than British participants did, and rape myth was a positive predictor of skills deficits. Step 2 explained significant incremental variance of 8%, Δ*F* (3, 677) = 20.74, *p* < .001. Machiavellianism was significantly and positively associated with skills deficits. Higher narcissism and psychopathy related to lower barriers based on skills (See Table 7).

**Table 7.** Hierarchical Regression Predicting Failure to Intervene Due to a Skills Deficit by Country, Gender, Rape Myths and Dark Triad.

	Beta (SE)	β	95% CI		*R*^2^
Step 1			Lower	Upper	
Intercept	3.57 (.19)				.07***
Gender (1 = female)	.65 (.16)	.17***	.09	.25	
Country					
Indonesia – U.K.	.14 (.16)	.09	−.11	.29	
Singapore – U.K.	.85 (.16)	.55***	.34	.75	
Rape myth total - log	1.15 (.48)	.11*	.2	.21	
Step 2					Δ*R*^*2*^
Intercept	5.38 (.41)				.08***
Gender (1 = female)	.58 (.15)	.15***	.07	.22	
Country					
Indonesia – U.K.	.31 (.16)	.20	−.01	.40	
Singapore – U.K.	.63 (.16)	.40***	.20	.61	
Rape myth total - log	1.39 (.47)	.14**	.05	.23	
Machiavellianism	.32 (.12)	.12**	.03	.21	
Narcissism	−.69 (.12)	−.24***	−.32	−.16	
Psychopathy	−.43 (.12)	−.15***	−.24	−.07	

*Note.* * *p* < .05, ** *p* < .01, *** *p* < .001*; CI= confidence interval*.

For failure to intervene due to audience inhibition, Step 1 was significant, with country and rape myths as unique predictors, *F* (4, 693) = 19.81, *p* < .001. Step 2 was significant, explaining 3% of the variance, Δ*F* (3, 690) = 8.10, *p* < .001. Similar to skills deficit, Machiavellianism was positively, while narcissism and psychopathy were negatively, associated with identifying an audience as a barrier to intervention (See Table 8).

**Table 8.** Hierarchical Regression Predicting Failure to Intervene Due to Audience Inhibition by Country, Gender, Rape Myths and Dark Triad.

	Beta (SE)	β	95% CI		*R*^2^
Step 1			Lower	Upper	
Intercept	2.33 (.28)				.10*
Gender (1 = female)	.22 (.13)	.06	−.01	.14	
Country					
Indonesia – U.K.	.30 (.16)	.19	.00	.39	
Singapore – U.K.	.51 (.16)	.32**	.12	.52	
Rape myth total - log	2.55 (.46)	.25***	.16	.34	
Step 2					Δ*R*^*2*^
Intercept	2.67 (.47)				.03*
Gender (1 = female)	.22 (.13)	.06	−.01	.14	
Country					
Indonesia – U.K.	.30 (.16)	.19	−.01	.39	
Singapore – U.K.	.33 (.16)	.21*	.01	.41	
Rape myth total - log	2.44 (.47)	.24***	.15	.33	
Machiavellianism	.44 (.12)	.16***	.07	.25	
Narcissism	−.39 (.12)	−.13***	−.21	−.06	
Psychopathy	−.26 (.12)	−.09*	−.17	−.01	

*Note.* * *p* < .05, ** *p* < .01, *** *p* < .001*; CI= confidence interval*.

For failure to notice, Step 1 was significant but only explained 5% of the variance, *F* (4, 693) = 9.68, *p* < .001. Only rape myths were significantly related to failure to notice, SE = 0.46, β = .19, 95% CI = 0.10, 0.28. The variance added in Step 2 was not significant, Δ*F* (3, 690) = 1.07, *p* = .36.

## Discussion

Our results indicated that the Dark Triad and rape myth acceptance relate to bystander barriers in a similar manner in Indonesia, Singapore, and United Kingdom, with only minor differences. In all three countries, Machiavellianism and rape myth acceptance were significant positive predictors of numerous perceived barriers to bystander intervention. Interestingly, narcissism and psychopathy related to fewer barriers, especially in relation to skills deficits and audience inhibition. When the shared variance between the Dark Triad and rape myth acceptance was taken into account, rape myth acceptance remained a significant positive predictor of multiple perceived barriers to active bystander intervention.

The differences between Machiavellianism and the two other Dark Triad traits are not surprising. Machiavellianism is characterised by predisposition to experience mental distress, high levels of anxiety, and elevated self-consciousness (Kowalski et al., 2019; Lyons, Evans, & Helle, 2019). Social anxiety has been associated with greater perceived barriers for intervening as a bystander in sexual assault situations (Uhrig, 2018). It could be that the more cautious and anxious nature of high Machiavellian individuals prevents them from intervening. The potential mediating/moderating factors (i.e., anxiety and cautiousness) between Machiavellianism and bystander barriers should be investigated further in future studies. Their relationship to the perpetrator may also be particularly important; given the long-term strategic nature of Machiavellianism, bystanders high on Machiavellianism may be reluctant to challenge those who may be of use to them in the future.

Interestingly, psychopathy and narcissism were related to lower self-reported skills deficits and audience barriers. The reduced audience barriers could be associated with high levels of social boldness and self-esteem (e.g. Miller et al., 2020), enabling intervention without a fear of negative evaluation from others. Psychopathy has also been linked to fearless heroism towards strangers (Smith et al., 2013), potentially facilitated by impulsivity and reduced physical and social fear (Murphy et al., 2017). This kind of heroism could be prominent in some situations of bystander actions, such as physically challenging a perpetrator in front of an audience. The relationship between narcissism and fewer skills deficits could also be explained by a generic over-estimation own abilities. Narcissism relates to illusions about one’s abilities and characteristics (e.g. attractiveness, intelligence and dominance; Gabriel et al., 1994; Grijalva & Zhang, 2016; Lyons, Blinkhorn, et al., 2019). The negative correlation with skills deficits as a barrier could relate to the self-enhancing style of narcissistic individuals, which could result in exaggeration of skills in self-report studies.

In addition to the Dark Triad, our research demonstrated the pervasive influence of rape myth acceptance as a barrier, irrespective of the country. Rape myth acceptance has been found to be a major bystander barrier in previous studies, mainly in the United States (e.g. Labhardt et al., 2017), with some evidence from Australia (Kania & Cale, 2018), Italy (Martini & De Piccoli, 2020) and Ecuador (Lyons, Brewer, et al., 2021). Our results showed that rape myth acceptance also prevents people from helping the victims in Indonesia, Singapore and the United Kingdom. This points to the importance of a victim empathy approach in bystander interventions. For example, one study found that an intervention programme aiming to increase men’s empathy towards rape victims was effective in reducing rape myths, increasing the willingness to intervene as a bystander (Langhinrichsen-Rohling et al., 2011). Our results suggest that these kinds of programmes could be useful in countries outside the United States too.

Interestingly, gender of the participant was a statistically significant predictor only when analysing skills deficits. Women, across the countries, reported higher skills deficits than men. Due to the relatively small number of men and low statistical power, we could not investigate gender as a moderator between the Dark Triad and barriers. The gender imbalance is unfortunately a common feature in psychology studies, due to convenience sampling of social sciences students. It is possible that narcissism and psychopathy relate to fewer skills deficits, but only in the men. Additionally, a previous study in a US sample found that higher levels of benevolent sexism in women (but not in men) correlated with skills deficits as a barrier (Yule et al., 2020). It would be interesting to investigate the links between gender, sexism, Dark Triad and skills deficits further in future research.

Our research is not without limitations. First, convenience/snowball sampling methods and the wording of the advertising somewhat limits the diversity of participants. We had a self-selected, young, mainly female university student sample, and the findings should not be generalised beyond this demographic group. Second, because the incidence of sexual violence on university campuses is well documented (e.g. Jozkowski & Wiersma-Mosley, 2017; Mkhize et al., 2020), the present study recruited university staff and students only. Globally, victims of sexual harassment are more likely to be women with low education levels (Cid & Leguisamo, 2021), highlighting the importance of research in non-university contexts (e.g., public spaces; Fileborn, 2017). Third, we did not take into consideration how the race or ethnicity of the victim relates to bystander barriers depending on the personality of the participant. For example, studies have found that white women are less likely to intervene when the victim is black (Katz et al., 2017). Knowing that the Dark Triad relates to racial prejudice (Koehn et al., 2019), it would be important to explore how these traits influence barriers when the victim is from another ethnic background. Fourth, the significant results had relatively low effect sizes, which somewhat tempers our enthusiasm regarding the implications of the results. However, even small effects can have some practical consequences (Gignac & Szodorai, 2016), and we do think that personality and bystander barriers are worthwhile further investigations. Finally, our research investigated different countries with diverse cultures using an etic, rather than an emic approach. It is possible that the structure of personality (e.g. Thalmayer et al., 2020), as well as the structure of bystander barriers are rather different outside the Northern American and Western European settings. It may be beneficial to employ a bottom-up, qualitative approach (e.g. Robinson et al., 2020) to investigate country-specific bystander barriers, as these may differ significantly depending on the complicated socio-cultural contexts.

In order to reduce sexual violence on campus, many universities have developed programmes designed to encourage bystander intervention. Bystander training can be effective in increasing the awareness, personal responsibility, attitudes and positive bystander behaviours (Mujal et al., 2021). In order to get maximum benefits from such intervention programmes, it is crucial to gain more knowledge of how personality x situation (e.g., Leone, Schipani-McLaughlin, et al., 2020) influences bystander barriers. Future studies should investigate methods for reducing barriers in individuals who possess rape myths, and have cynical, manipulative personality traits.

In summary, our findings suggest that the relationship between bystander barriers, personality, and rape myths are similar in three different countries (Indonesia, Singapore, and United Kingdom). Machiavellianism and rape myth acceptance related to diverse bystander barriers, and narcissism and psychopathy were associated with reduced skills deficits and audience inhibition. Deeper understanding of the relationships between personality and barriers to helping could have important future applications in helping to devise strategies that encourage active bystander behaviours.

## Appendix 1

### Differences in Perceived Barriers, Rape Myths, and Dark Triad across Countries

To examine barriers to bystander intervention, we compared the three countries on levels of reported barriers in a MANCOVA, controlling for gender. The MANCOVA was significant, Wilk’s Lambda = .77, *F* (10, 1390) = 19.47, *p* < .001. All univariate tests were significant across the types of barriers, including identifying risk, taking responsibility, skills, and audience inhibition and failure to notice, *F*s (2699) = 17.69, 83.44, 16.54, 24.58 and 8.59 respectively (all *p*-values <.001). As shown by the descriptive statistics in Tables 1–3, levels in the UK were lower than Singapore and Indonesia. Gender was also significant, Wilk’s Lambda = .95, *F* (5, 695) = 8.15, *p* < .001. Differences were significant in taking responsibility, *F* (1,699) = 9.54, *p* = .002) and skills, *F* (1,699) = 13.52, *p* <.001). Men (*M* = 3.51, *SD* = 1.06) reported that taking responsibility was more of a barrier than for women (*M* = 3.00, *SD* = 0.98). Yet, women (*M* = 4.63, *SD* = 1.54) reported that skills were more of a barrier to intervention than men (*M* = 4.32, *SD* = 1.64). Because we did not expect differences across the individual barriers by country, we did not conduct further ANOVAs or independent-samples t-tests. Also, we did not have enough power to look at interactions between gender and country.

Differences in reports of rape myths were examined with an ANCOVA while controlling for gender. We log transformed the variable to normalize it. There was a significant difference across the countries, *F* (2,682) = 149.80, *p* < .001, η^2^ =.29 and gender, *F* (1,682) = 59.00, *p* < .001, η^2^ =.06. Men (*M* = 2.31, *SD* = .77) were higher than women (*M* = 1.72, *SD* = 0.61), when examining the non-transformed means. Post-hoc tukey tests showed that Indonesia was higher than Singapore, *t* (682) = 5.11, *p* < .001, *d* = .56, which was higher than the U.K., *t* (682) = 9.30, *p* < .001, *d* = .96. The descriptive statistics are shown in Tables 1–3.

To examine the levels of Dark Triad traits across countries, we conducted another MANCOVA. Wilk’s Lambda was significant, .72, *F* (6, 1396) = 40.80, *p* < .001, with significant differences in Machiavellianism, narcissism, and psychopathy, *F*s (2, 700) = 91.07, 42.18, 41.17, respectively (*p*s < .001). Gender was significant, such that men and women differed in Dark Triad traits, Wilk’s Lambda = .96, *F* (4, 698) = 10.60, *p* < .001; differences were significant in Machiavellianism, *F* (1,700)=15.30, *p* < .001, and psychopathy, *F* (1,700) = 27.91, *p* < .001), but not narcissism, *F* (1,700) = 2.21, *p* = .137). Men were higher on Machiavellianism (*M* = 3.32, *SD* = .61) and psychopathy (*M* = 2.48, *SD* = 0.59) than women (*M* = 3.01, *SD* = .57; *M* = 2.20, *SD* = .54, respectively). As seen in the descriptive statistics (Tables 1–3), Indonesia showed higher levels across the Dark Triad, with the U.K. and Singapore more similar in narcissism and psychopathy levels.

In sum, men and women differed on many of the barriers, Dark Triad and rape myths. Country level differences were also evident, particularly for barriers for intervention in sexual harassment, Dark Triad traits, and endorsement of rape myths. In particular, levels in Indonesia were generally reported to be higher across all of these.
